# Local Reports of Epidemic and Endemic Diseases

**Published:** 1855-09

**Authors:** 


### LOCAL REPORTS OF EPIDEMIC AND ENDEMIC DISEASES.

*During the Months of June, July, and August* 1855.

THE subjoined Report is compiled from data furnished specially for this journal by medical men in seventeen towns in England and Wales, viz.:

| Place. | County. | Lat. | Long. | Observer. |
|---|---|---|---|---|
| Bridgewater - | Somerset - | 51. 8 N. | 3. 0 E. | Alfred Haviland, Esq. |
| Canterbury - | Kent - - | 51.17 N. | 1. 4 E. | G. Rigden, Esq. |
| Chatham - | Kent - - | 51.24 N. | 0.14 E. | F. J. Brown, M.D. |
| Up. Holloway | Middlesex - | 51.32 N. | 0.03 W. | W. B. Kesteven, Esq. |
| Swansea - | Glamorgansh. | 51.38 N. | 3.50 W. | W. H. Michael, Esq. |
| Saffron Walden | Essex - - | 52. 3 N. | 0.12 E. | H. Stear, Esq. |
| Bedford - - | Bedfordshire - | 52. 8 N. | 1.51 W. | T. H. Barker, M.D. |
| Thetford - | Norfolk - - | 52.26 N. | 0.45 E. | H. W. Bailey, Esq. |
| Wisbeach - | Cambridgesh. | 52.40 N. | 0.10 E. | W. H. Hole, Esq. |
| East Dereham | Norfolk - - | 52.40 N. | 0.57 E. | J. Vincent, M.D. |
| Pontesbury - | Shropshire - | 52.43 N. | 2.50 W. | Wm. Eddowes, Esq. |
| Nottingham - | Nottinghamsh. | 52.57 N. | 1.10 W. | T. Robertson, M.D. |
| Hawarden - | Cheshire - | 53.11 N. | 3. 2 W. | T. Moffat, M.D. |
| Alford - - | Lincolnshire - | 53.15 N. | 0. 6 E. | R. U. West, Esq. |
| Gainsborough | Lincolnshire - | 53.23 N. | 0.47 W. | D. Mackinder, M.D. |
| Liverpool - | Lancashire - | 53.24 N. | 2.59 W. | Thos. Bickerton, Esq. |
| Bolton - - | Lancashire - | 53.35 N. | 2.19 W. | W. H. Pendlebury, Esq. |

### QUARTERLY STATEMENT—No. III.

| Disease. | Where Observed. | At what Periods.—Weeks Ending |
|---|---|---|
| 1. Scarlet Fever - | Bridgewater - | July 6 |
| " | Canterbury - | June 8, 15, 22 |
| " | Chatham - | June 8; Aug. 17, 24 |
| " | Swansea - - | June 8 to July 6 |
| " | Saffron Walden | June 15; July 13; Aug. 10, 17, 24 |
| " | Bedford - | Entire Quarter |
| " | Thetford - - | June 8, 22; July 27; Aug. 17, 24, 31 |
| " | Wisbeach - | July 13, 20, 27 |
| " | Pontesbury - | June 8, 15 |
| " | Nottingham - | June 8, 15, 22; July 20, 27; Aug. 3, 24, 31 |
| " | Hawarden - | June 8, 15; Aug. 17 |
| " | Liverpool - | Every week |
| " | Bolton-le-Moors | Every week |
| 2. Measles - - | Canterbury - | July 6, 13 |
| " | Chatham - - | Every week |
| " | Swansea - - | All June and July, and Aug. 10 |
| " | Bedford - | Aug. 10, 17, 24 |
| " | Thetford - - | June 8, 15, 29; all July; Aug. 10, 24, 31 |
| " | East Dereham - | July 13, 20; Aug. 23, 31 |
| " | Pontesbury - | June 22, 29; July 6 |
| " | Nottingham - | June 15; July 20 |
| " | Hawarden - | June 8, 15; Aug. 17 |
| " | Alford - - | Aug. 3 |
| " | Liverpool - | Every week |
| " | Bolton-le-Moors | Every week |

| Disease. | Where Observed. | At what Periods.—Week Ending |
|---|---|---|
| 3. Small-pox - | Canterbury - | Every week |
| " | Chatham - - | June 8, 29; July 6 |
| " | Upper Holloway | July 29 |
| " | Bedford - - | July 6 to Aug. 10 |
| " | Nottingham - | June 8; July 6 |
| " | Liverpool - | Every week except June 22; Aug. 3, 24 |
| " | Bolton - - | June 22, 29; July 6; Aug. 3, 10, 17 |
| 4. Hooping-cough | Canterbury - | Aug. 10 to Aug. 31 |
| " | Chatham - - | July 27 |
| " | Swansea - - | June 8 to Aug 10 |
| " | Saffron Walden | July 27 to Aug. 31 |
| " | Bedford - - | Aug. 10, 17, 24 |
| " | Thetford - - | July 6, 13, 20; Aug. 3 to Aug. 31 |
| " | Wisbeach - | Aug. 24, 31 |
| " | East Dereham | Aug. 17, 24, 31 |
| " | Pontesbury - | Every week |
| " | Nottingham - | June 8, 15, 29; July 13 to Aug. 10; 31 |
| " | Gainsborough - | July 27 to Aug. 31 |
| " | Liverpool - | June 29; July 6, 13; Aug. 17, 31 |
| " | Bolton - - | July 6 to Aug. 10 |
| 5. Croup - - | Chatham - - | June 15; July 6 |
| " | Swansea - - | June 15; July 6 |
| " | East Dereham | June 15; Aug. 17 |
| " | Nottingham - | June 8 to July 13; July 27 to Aug. 17 |
| " | Alford - - | June 15 |
| " | Gainsborough - | Aug. 31 |
| " | Liverpool - | June 22, 29; July 6; Aug. 24, 31 |
| " | Bolton - - | June 22, 29 |
| 6. Catarrh - - | Bridgewater - | June 8, 22; July 20; Aug. 17 |
| " | Chatham - - | June 15, 22; July 13 to Aug. 31 |
| " | Upper Holloway | July 15 |
| " | Swansea - - | June 15 to July 6 |
| " | Saffron Walden | July 6 |
| " | Thetford - - | June 15, 22; July 13, 27; Aug. 10 |
| " | Wisbeach - | Aug. 10 to Aug. 31 |
| " | Pontesbury - | June 8 |
| " | Nottingham - | June 8; July 6 to Aug. 24 |
| " | Hawarden - | July 20; Aug. 3, 24 |
| " | Alford - - | June 8; July 6 |
| " | Gainsborough - | Every week |
| " | Liverpool - | July 8, 22, 29; July 13, 20; Aug. 24, 31 |
| " | Bolton - - | June 8 to July 13; Aug. 3 to Aug. 31 |
| 7. Influenza - | Bridgewater - | July 13, 27; Aug. 10, 17, 31 |
| " | Swansea - - | June 8 to July 27; Aug. 24, 31 |
| " | Thetford - | June 8, 22, 29; July 27; Aug. 3 |
| " | Nottingham - | Aug. 3, 10 |
| " | Alford - - | June 22 |
| " | Gainsborough - | June 8, 15 |
| " | Liverpool - | June 15; Aug. 31 |
| " | Bolton - - | July 20, 27 |
| 8. Erysipelas - | Canterbury - | July 13, 20; Aug. 31 |
| " | Chatham - - | June 22, 29; July 6, 20, 27; Aug. 3, 31 |
| " | Saffron Walden | June 8, 29 |
| " | Bedford - - | June 29; July 6 |
| " | Thetford - - | June 8, 22, 29; Aug. 3, 10, 24 |
| " | Wisbeach - | June 22, 29 |
| " | Pontesbury - | June 22, 29; July 27; Aug. 3. |

| Disease. | Where Observed. | At what Periods.—Weeks Ending |
|---|---|---|
| 8. Erysipelas - | Nottingham - | June 8, 15, 22; July 13, 20, 27; Aug. 3 |
| " | Alford - - | June 22 |
| " | Gainsborough - | Aug. 31 |
| " | Liverpool - | June 8, 15; July 6, 20, 27 |
| " | Bolton - - | July 27 to Aug. 31 |
| 9. Cholera (Eng.) | Chatham - - | Aug. 10, 24, 31 |
| " | Liverpool - | July 6, 27; Aug. 17, 24, 31 |
| " | Bolton - - | July 20, 27; Aug. 3, 24, 31 |
| 10. Ague - - | Canterbury - | July 6, 13; Aug. 17 |
| " | Chatham - - | June 8; June 29 to July 27; Aug. 10, 24 |
| " | Upper Holloway | July 22 |
| " | Thetford - - | June 15; July 13, 27; Aug. 17 |
| " | Wisbeach - | June 8, 15, 22; July 20, 27; Aug. 17, 24, 31 |
| " | East Dereham | June 29 to Aug. 3 |
| " | Alford - - | July 27; Aug. 3 |
| " | Liverpool - | Aug. 17 |
| 11. Remittent Fever | Canterbury - | July 6, 13, 20; Aug. 10 |
| " | Chatham - - | June 15; July 6, 13, 27; Aug. 3, 24, 31 |
| " | Upper Holloway | July 8, 15 |
| " | Thetford - - | June 8, 22, 29; July 6; Aug. 10, 24, 31 |
| " | Wisbeach - | July 13, 20 |
| " | East Dereham | July 6 to Aug. 3 [17, 31 |
| " | Nottingham - | June 15; June 29 to July 27; Aug. 10, |
| " | Liverpool - | July 13 |
| " | Bolton - - | June 8, 15, 22, 29 |
| 12. Diarrhœa - | Bridgewater - | Every week |
| " | Canterbury - | June 22 to Aug. 31 |
| " | Chatham - - | Every week |
| " | Upper Holloway | July 8, 29; whole of August |
| " | Swansea - - | Every week [24, 31 |
| " | Saffron Walden | June 22, 29; July 6, 20, 27; Aug. 3, 10, |
| " | Bedford - - | July 6, July 20 to Aug. 31 [Aug. 31 |
| " | Thetford - - | June 8, 15, 29; July 6, 13; July 27 to |
| " | Wisbeach - | Aug. 3 to Aug. 31 |
| " | East Dereham | July 13 to Aug. 31 |
| " | Pontesbury - | June 15, 22, 29; July 6; Aug. 17, 24, 31 |
| " | Nottingham - | Every week |
| " | Hawarden - | Every week except Aug. 24 |
| " | Alford - - | June 29; July 6, 27; Aug. 10, 17 |
| " | Gainsborough - | June 8 to July 20; Aug. 24, 31 |
| " | Liverpool - | Every week except June 22 |
| " | Bolton - - | July 6 to Aug. 31 |
| 13. Dysentery - | Bridgewater - | July 13 |
| " | Canterbury - | Aug. 24 |
| " | Chatham - - | July 27 to Aug. 31 |
| " | Swansea - - | June 29; July 6 |
| " | Bedford - - | Aug. 3, 10, 17 |
| " | East Dereham | June 15, 22 |
| " | Pontesbury - | June 29; July 6 |
| " | Nottingham - | June 8 to July 20; Aug. 10 to 31 |
| " | Alford - - | June 22, 29 |
| " | Gainsborough - | June 29 |
| " | Liverpool - | July 20 |
| " | Bolton - - | June 22, 29; July 3; Aug. 3 to 31 |
| 14. Typhus - - | Canterbury - | June 8; June 29 to July 27; Aug. 17, 24, 31 |
| " | Chatham - - | June 8, 15; July 20 to Aug. 17; Aug. 31 |
| " | Swansea - - | June 22, 29; July 6 |

| Disease. | Where Observed. | At what Periods.—Weeks Ending |
|---|---|---|
| 14. Typhus - - | Bedford - - | July 20 to Aug. 31 |
| " | Thetford - - | June 8, 29; July 6, 20; Aug. 17 |
| " | East Dereham - | Aug. 3 to Aug. 31 |
| " | Pontesbury - | June 8, 15 |
| " | Nottingham - | June 8, 22, 29; July 13; Aug. 3, 31 |
| " | Hawarden - | June 22 |
| " | Gainsborough - | Every week except Aug. 31 |
| " | Liverpool - | July 6, 13, 20 |
| " | Bolton - - | July 13, 20 |
| 15. Puerperal Fever | Bridgewater - | June 8 |
| " | Nottingham - | June 8 |
| " | Hawarden - | Aug. 24 |
| " | Alford - - | June 15, Aug. 3 |
| " | Bolton - - | July 20, 27 |
| 16. Carbuncle - | Bridgewater - | (Furunculi) Every week [24, 31 |
| " | Canterbury - | June 8,15,22,29; July 13,20; Aug. 17, |
| " | Saffron Walden | July 27; Aug. 10, 31 |
| " | Wisbeach - | June 8 |
| " | Pontesbury - | June 22,29; July 20; Aug. 10,17,24,31 |
| " | Nottingham - | July 13, Aug. 10 |
| " | Alford - - | June 29 |
| " | Liverpool - | July 13; Aug. 3, 10, 31 |
| " | Bolton - - | June 22; July 27; Aug. 3 |

### ADDITIONAL OBSERVATIONS.

*Bridgewater.*—Mr. Haviland furnishes a continuation of his medico-meteorological observations.

"*June* 8th.—Ozone, 42. Wind S.W. S.E. Range of barometer, 0·23 in. Of the thermometer, 17°; highest, 59°; lowest, 42°.

"Deaths from disease of lungs, 2; puerperal peritonitis, 1; sudden death, 1; tabes mesenterica, 1; total, 5.

"— 15.—Ozone, 38. Wind var. Range of barometer, 0·90 in. Range of thermometer, 14°; highest, 69°; lowest, 55°.

"Deaths from peritonitis, 1; hydrocephalus, 1; total, 2.

"— 22.—Ozone, 19. Wind N.W. Range of barometer, 0·25 in. Of thermometer, 7°; highest 59°; lowest 52°.

"Deaths from disease of heart, 1; inflammation of lungs, 1; chronic hepatitis, 1; hydrocephalus, 1; total, 4.

"— 29.—Ozone, 8. Wind N.W. E. and S.E. Range of barometer, 0·45 in. Of thermometer, 14°; highest, 72°; lowest, 58°.

"Deaths from hydrocephalus, 1; consumption, 1; total, 2.

"*July* 6.—Ozone, 8. Wind S.W. N.W. and E. Range of barometer, 0·30 in. Of thermometer, 8°; highest, 69°; lowest, 61°.

"Deaths from paralysis, 1; senile gangrene, 1; fever, 1; apoplexy, 1; convulsions, 1; congestion of the brain, 1; total, 6.

"— 13.—Ozone, 24. Wind E. and N.W. Range of barometer, 0·54 in. Of thermometer, 11°; highest, 72°; lowest, 61°.

"Deaths from consumption, 2; chronic disease of liver, 1; total, 3.

"— 20.—Ozone, 35. Wind N.W. Range of barometer, 0·40 in. Of thermometer, 6°; highest, 66°; lowest, 60°.

"Deaths from consumption, 3; disease of the lungs, 1; marasmus, 1; total, 5.

"— 27.—Ozone, 4. Wind N.W. Range of barometer, 0·38 in. Of thermometer, 3·5°: highest, 63·5°; lowest, 60°.

"Deaths from ch. bronchitis, 1; fever, 1; pneumonia, 1; total, 3.

*August* 3.—Ozone, 18. Wind S.W. Range of barometer, 0·18 in. Of thermometer, 6°; highest, 67°; lowest, 61°.

"Deaths from unknown drowning, 1; chronic disease of the bowels, 1; strumous abscess, 1; consumption, 1; convulsions, 1; total, 5.

"— 10.—Ozone, 29. Wind N.W. Range of barometer, 0·33 in. Of thermometer, 4°; highest, 63°; lowest, 59°.

"Death from dysenteric diarrhœa, 1; total, 1.

"— 17.—Ozone, 14. Wind N.W. Range of barometer, 0.10 in.?

"Deaths from marasmus, 2; synochus and peritonitis, 1; phthisis, 1; ulceration of the bowels, 1; total, 5.

"— 24.—Ozone, 0. Wind N.W.

"Deaths from convulsions, 1; drowning, 1; total, 2.

"— 31.—Ozone, 6. Wind N.W. Range of barometer, 0·48 in. Of thermometer, 9°; highest 65°; lowest, 56°.

"Deaths from disease of heart, 2; exhaustion, 1; diarrhœa, 1; total, 4.

"Neuralgia, rheumatism, and boils, have been most prevalent during this quarter. Vegetation was very backward at first. Wheat first appeared in ear about June 9th, and was almost universally in blossom about the 28th. Hay-making did not commence until about June 21st, and harvesting about August 9th. Oats were cut a little earlier. August 7th.—Insects of all descriptions most abundant. The potato disease appeared, about July, to be rather prevalent. Apples abundant. The corn was much laid by the heavy thunder-storms which took place June 29th, July 13th and 19th. The autumnal tints appeared very early—about the last fortnight of August. The turnips affected much by the fly."

*Canterbury.*—Mr. Rigden states that, "Diarrhœa, although occurring in most weeks during the past quarter, has not assumed a malignant character. Small-pox has continued to spread; and a large proportion—about one-third of the cases that have been brought under my observation—have been after vaccination; but these cases have mostly been of a milder character, and with little or no secondary fever. In the epidemic of this disease, occurring in 1837-8, but one case in sixteen had been vaccinated; in another epidemic, occurring in 1844-5, of thirty-three cases, but one had been vaccinated; in an epidemic, occurring in 1847-8, one in four had been vaccinated."

*Chatham.*—Dr. Brown writes: "Vegetation has been healthy and luxuriant during the past quarter. The growth of fruit has been very great, and the harvest is abundant. The hops are extraordinarily rich, clean, and heavy. Many dogs have been affected with rabies; and a whole flock of sheep was destroyed by a farmer in the vicinity, in consequence of the difficulty of distinguishing betwixt the sheep that were bitten and those not so.

Dysentery has prevailed during the latter half of the quarter. The cases have been exceedingly severe, and several deaths have occurred. Diarrhœa has been prevalent throughout the quarter. I know of five cases of sporadic Asiatic cholera. One proved fatal. Measles have occurred in great number, and have been succeeded by fatal thoracic and abdominal sequelæ. Diphtheritis has occurred as one of the sequelæ. One case of purpura has been observed. There have been cases of furunculus and of cellulitis, but none of anthrax."

*Swansea.*—Mr. Michael states: "That the period embraced in this report has been extremely healthy; hardly any disease of consequence prevailing."

*Saffron Walden.*—Mr. Stear writes: "Scarlatina has been prevalent during the past quarter. I have had three fatal cases: the general type of the disease has been of the simple form. Cases of hooping-cough have also been frequent. The cases of diarrhœa have been isolated, but numerous."

*Bedford.*—Dr. Barker writes: "Scarlet fever has prevailed in Bedford and the neighbourhood during the entire quarter, and the mortality has been considerable. Cases of the mildest and of the most malignant character have occurred, and the mortality has by no means been confined to the poorer classes, or to the most unsanitary localities. Death has occurred; in a few cases, from prostration coming on in a few hours before the rash had made its appearance. The sequelæ of the disease have been numerous, and in many cases fatal. Among them have occurred cases of anasarca, albuminuria, diseases of the ear and eye, suppuration of the cervical glands, and hæmaturia. In a few cases measles and scarlatina have occurred together in the same individuals."

*Thetford.*—Mr. Bailey writes: "In my report for the quarter, I must first premise some medico-meteorological observations.

"During the month of June, we have not recorded so dry a month for many years in this locality; the amount of rain being only an inch; the greater part falling on the 7th and 15th. The prevalent winds, S.W. Range of the barometer, an inch. Range of thermometer for the month, 37°: maximum, 83°; minimum, 46°. Scarlatina still continues amongst us, only two or three cases coming under observation; they are of mild character, and in a few days are convalescent. From the 15th to the 22nd a great reduction of temperature took place, which produced many cases of catarrh, influenza, bronchitis in children, diarrhœa, one case of typhus, and two of remittent fever. Erysipelas also came under notice, and might be considered epidemic. From this period to the end of the month the temperature increased, when diarrhœa became more prevalent.

"July was a wet month, the rain fallen was three inches; prevalent winds, N.W.; range of the barometer, 7-10ths of an inch. The thermometer was exceedingly fluctuating: maximum, 82°; minimum, 54°; range, 28°. No case of scarlatina has occurred during the month, but measles are becoming more prevalent amongst children, and some cases in adults. No fatal cases have occurred. Hooping cough follows measles: some cases are very severe, requiring active treatment. Two cases of diarrhœa of a severe kind came under medical treatment. A fatal case of uterine hæmorrhage in a woman of fifty, which resisted every means of medical treatment. Two cases of atrophy in children, and a case of erysipelas in a woman, who had been in a hospital and attacked with it. Those diseases, uninfluenced by atmospheric causes, and under medical treatment are icterus, hydrocephalus (fatal), ascites, neuralgia, convulsions, and amenorrhœa.

"August. The temperature of this month is only two degrees above summer heat, not exceeding 78°, and the lowest 54°. Range, 24°; range of barometer, 7-10ths of an inch. Prevailing winds, S.W. With the exception of scarlatina, which has become more prevalent, and measles, with hooping cough, the month may be considered very healthy—indeed the whole district from the registrar's book; the deaths amounting to 38. From the high temperature diarrhœa is daily under treatment; one case proved fatal in a child four years old.

"There has been no epidemic among cattle, with the exception of some few inflammatory affections. The harvest, although late, seems to promise an average crop. Potatoes are more free from disease than for many years; there is a promise of an abundant rise.

"The following is the number of cases from the registrar's book of deaths:

| *June.* | | *July.* | | *August.* | |
|---|---|---|---|---|---|
| Debility in new-born children | 5 | Atrophy in infants | 2 | Pulmonary consumpt. | 3 |
| Pulmonary consumpt. | 1 | Inflammation of chest | 2 | Pertussis | 1 |
| Cancer | 1 | Debility | 3 | Fever (typhoid) | 1 |
| Atrophy in an infant | 1 | Encephalitis | 1 | Phlebitis | 1 |
| Encephalitis | 1 | Uterine hæmorrhage | 1 | Diarrhœa | 1 |
| Ascites | 1 | Pulmonary consumpt. | 5 | | |
| Decay of nature | 2 | Hydrocephalus | 1 | | |
| Marasmus | 1 | Enteritis | 1 | | |
| Inflammation of chest | 1 | | | | |
| Hydrothorax | 1 | | | | |
| | 15 | | 16 | | 7 |

Total, 38.

*Wisbeach.*—Mr. Hole states that, "Intermittent has prevailed to a greater extent this spring and summer than for many previous years, owing, probably, to the severity of the east wind, which continued up to the month of June."

*East Dereham.*—Dr. Vincent says that the "measles occurring in July were imported from Norwich. The remittent fever occurred in a child from Lynn Regis, a marshy district. Both cases of croup were fatal. Disease shows itself in the potato. The wheat also is affected with what the farmers term 'blight.' The kernel is very much shrivelled."

*Pontesbury.*—Mr. Eddowes says that "measles and scarlet fever, as well as hooping cough, were very prevalent in May in this district.

*Nottingham.*—Dr. Robertson writes: "The past quarter has been, generally speaking, exceedingly healthy. A glance at the returns will show that some epidemic diseases have been almost, if not entirely, absent. Amongst these are measles, small-pox, influenza, cholera, ague, puerperal fever. Carbuncle, hooping-cough, croup and catarrh have all been general, though not extensively diffused. Whether any connexion exists between the occurrence of these respiratory diseases and the temperature of the quarter, which in June was $2\frac{1}{2}°$ less than the mean of 42 years, and in July and August also less than the mean, though not to so great an amount, cannot with the present data be conclusively asserted. By reference to Mr. Lowe's register, I find that in June the range of temperature was 43·5, in July 38·3, in August 40·5. In June the mean amount of ozone was 4·7 by night and 4·8 by day; in July it was 2·9 by night and 4·6 by day; and in August the mean was by night 3·5 and by day 3·9. Rain fell on 13 days in June, 13 in July, and 14 in August. To show the lateness of the season the following facts are interesting: The lilac was in bloom 17 days, the laburnum 14, the hawthorn 17, and the acacia 8 days later than usual. Strawberries were ripe 17 days, currants 6 days, raspberries 15, apricots 12, and barley 9 days later. On the 31st of May there was a great mortality amongst the swallows from extreme cold; about the 31st of August the potato disease showed itself and soon became general; and at the same time the vine disease appeared. Partridges have, many of them, a curious disorder, consisting of wart-like excrescences about the head. They are otherwise strong and in good condition. No disease has been epidemic amongst cattle, and no respiratory diseases (for the time of year) have been unusually prevalent. I subjoin a table of diseases and deaths (the former obtained from several practitioners in the town, and the latter from the registrar's returns), which will show at a glance the comparative commencement of disease, and the mortality of the town for the past quarter. I may add that the corresponding total number of deaths for last year's similar quarter was 411. Many deaths occur here under two years of age; and on this subject I may express my coincidence with a paragraph from an admirable report issued by the sanitary committee in the year 1854, which remarks that 'This number of deaths (which largely influences the average rate of mortality) prevails mainly among the children of the operative classes; and while the great importance of attention to improving and purifying the most densely inhabited parts of the town is thus strongly shown, an improved result must depend mainly on judicious maternal management, and on the better ventilation and greater cleanliness of the dwelling houses of the poor.'

"Number of diseases observed to commence and deaths occurring during the quarter:

| Week ending | | Diseases. | Deaths. |
|---|---|---|---|
| June | 8 | 74 | 19 |
| „ | 15 | 68 | 25 |
| „ | 22 | 60 | 25 |
| „ | 29 | 77 | 28 |
| July | 6 | 73 | 20 |
| „ | 13 | 66 | 18 |
| „ | 20 | 59 | 20 |
| „ | 27 | 69 | 14 |
| August | 3 | 65 | 27 |
| „ | 10 | 66 | 22 |
| „ | 17 | 59 | 24 |
| „ | 24 | 56 | 20 |
| „ | 31 | 69 | 24 |
| | Total | 861 | 286 |

"Out of the 861 cases of disease of which the commencement was noted as above stated, 487 were zymotic, and 281 were cases of diarrhœa, &c. (epidemics peculiar to the quarter). I can quite bear out Dr. Barnes's remark, that the 'diarrhœa of the present season is a very different affection from the choleraic diarrhœa of last autumn'; and I would refer to his lucid and excellent paper, 'On the registration of prevailing diseases', in the *Lancet* of Sept. 8, as illustrating, in a most convincing manner, the value of such inquiries in the field of sanitary science as the Editor of this Journal has lately undertaken.

*Alford.*—Mr. West says: "Abortions within the first three months of gestation have been unusually frequent during the past quarter, attended with very great hæmorrhage. A case of hooping-cough was imported. Low or asthenic pneumonia was present June 8th, 15th, 22nd, and 29th."

*Gainsborough.*—Dr. Mackinder remarks: "I have had but one case of croup, which was fatal. It happened to a delicate little girl, to whom I was summoned four days after the disease had shown itself. Also but one case of typhus, which is now convalescent, though the recovery has been very protracted; the patient residing in a dirty ill-ventilated yard. Two or three cases of influenza have come under my observation; one of erysipelas; one of dysentery; a few of catarrh and diarrhœa; and half-a-dozen cases of hooping-cough among the children in the workhouse. Some of the latter cases of diarrhœa have assumed the more painful form of English cholera, being accompanied with cramps and vomiting. Gastro-enteritis was very prevalent during the first half of the quarter.

"This quarter has been a very healthy one. Mr. Dyson has favoured me with the following meteorological report. 'The quarter ending 31st August has been remarkable chiefly for the excess of rain amounting to 5·08 inches, which fell in July; of which, $1\frac{1}{2}$ in. fell on the 14th, during a violent storm of thunder and lightning. Only 2 inches fell in June, and scarcely 1 in August, making a total of 8·08 inches in the quarter, rather more than the average. The highest temperature in June reached 84° on the 6th; in July, 82° on the 13th; in August, 78° on the 18th. The lowest in June, 41° on the 23rd; in July, 46° on the 6th; in August, 48° on the 9th. The average barometrical pressure was very nearly thirty inches throughout the quarter. Rain fell on thirty-six days. Violent storms of thunder, lightning, and rain occurred on June 8th, 9th, and 15th, and on the 14th, 19th, 24th, 25th, and 27th of July. The amount of ozone developed was small throughout the three months. The first wheat was cut on August 18th, and general on the 20th; being nearly a fortnight later than usual."

*Liverpool.*—Mr. Bickerton remarks "that in the Toxteth Park District, diarrhœa was very prevalent during August. In the North Town District, measles were very extensive during the third week of June, and diarrhœa and malignant scarlet fever in the month of August. One case of farcy and glanders occurred and terminated fatally on August 14th."

*Bolton-le-Moors.*—Mr. Pendlebury observes: "Scarlet fever and measles have been most prevalent. The former, in the majority of cases, was of the simple type; but in most dropsy was a sequela, and especially so since the mornings and evenings have become chilly. No cases of malignant cholera have to my knowledge occurred; such that have been prevailing have been chiefly of choleraic diarrhœa, brought on by error of diet. We have not had the usual amount of diarrhœa. There have been two cases of erratic erysipelas in suckling infants. One occurred in my own practice, in a child aged two months. It was first perceptible on the day following that of its being vaccinated, and continued wandering in its character till its subsidence on the fourteenth day, when the fever (which had kept the vaccinia in subjection) also abated, and allowed of the forming of the vaccine vesicle, which in two to three days became fully matured. Cases of neuralgia and neuralgic rheumatism have been rather frequent. The rate of mortality has, during the past quarter, been below the average."

